# Lung Cancer Risk Factors Beyond Smoking in Ethiopia: A Multicenter Matched Case–Control Study

**DOI:** 10.3390/cancers18060914

**Published:** 2026-03-12

**Authors:** Nathan Estifanos, Gudina Egata, Adamu Addissie, Rahel Argaw Kebede, Aschalew Worku, Amsalu Bekele, Biruk Habtamu, Selam Tesfaye, Aman Yesuf Endries, Zemzem Shigute, Anagaw Derseh Mebratie, Getnet Alemu, Arjun S. Bedi, Negussie Deyessa

**Affiliations:** 1 School of Public Health, College of Health Sciences, Addis Ababa University, Addis Ababa P.O. Box 9086, Ethiopia; gudina.egata@aau.edu.et (G.E.);; 2 College of Medicine and Health Science, Wollo University, Dessie P.O. Box 1145, Ethiopia; 3 School of Medicine, College of Health Sciences, Addis Ababa University, Addis Ababa P.O. Box 9086, Ethiopia; 4 Oncology Department, School of Medicine, College of Health Sciences, Haramaya University, Harar P.O. Box 235, Ethiopia; 5 Department of Oncology, Faculty of Medical Sciences, Institute of Health, Jimma University, Jimma P.O. Box 378, Ethiopia; 6 School of Public Health, St Paul’s Hospital Millennium Medical College, Addis Ababa P.O. Box 1271, Ethiopia; 7 International Institute of Social Studies (ISS), Erasmus University Rotterdam, P.O. Box 29776 The Hague, The Netherlands; 8 Institute of Development and Policy Research, Addis Ababa University, Addis Ababa P.O. Box 1176, Ethiopia

**Keywords:** lung cancer, risk factors, case–control study, Ethiopia

## Abstract

Background: While smoking is the dominant global driver of lung cancer, less than a quarter of Ethiopian patients have ever smoked, pointing to locally relevant risk factors. Evidence to guide prevention and early detection in resource-limited settings is scanty. Methods: To address this gap, we conducted a multicenter matched case–control study including 351 histopathologically confirmed primary lung cancer cases and 702 hospital-based controls matched by sex, age (±5 years), and residence. Directed acyclic graphs informed the selection of variables, and multivariable hierarchical conditional logistic regression was used to identify risk factors beyond smoking. Results: The analysis shows that lung cancer was independently associated with low education, wealth, solid-fuel use, occupational exposure, insufficient physical activity, meat-based and processed food dietary patterns, secondhand smoke (SHS), prior tuberculosis, and family history of cancer. Subgroup analysis by sex revealed consistent associations across males and females, but exposure distributions explained sex-specific patterns: smoking, occupational exposure, meat-based diets, and family history were more common among males, whereas SHS, the use of solid fuels, and processed food dietary patterns predominated in females. Conclusions: Lung cancer in Ethiopia appears to be associated with several factors in addition to smoking. Gender-sensitive public health interventions targeting these locally relevant risk factors are essential for effective prevention and early detection.

## Simple Summary

In Ethiopia, smoking explains only a fraction of lung cancer cases, highlighting the importance of other local risk factors. This study examined 351 lung cancer patients and 702 matched hospital controls to uncover these drivers. Beyond smoking, lung cancer was independently associated with low education and wealth, use of solid fuels, occupational exposures, limited physical activity, processed food- and meat-based dietary patterns, secondhand smoke, prior tuberculosis, and family history of cancer. While both men and women were affected by these risks, men were more exposed to smoking, occupational hazards, meat-based diets, and family history. In contrast, women faced greater exposure to secondhand smoke, solid fuels, and processed food diets. These findings underscore the need for gender-sensitive public health strategies that address multiple local risk factors to improve lung cancer prevention and early detection in resource-limited settings like Ethiopia.

## 1. Introduction

Lung cancer remains one of the leading causes of cancer incidence and mortality [1,2]. Ongoing sociodemographic and epidemiological transitions are contributing to the cancer burden in low- and middle-income countries (LMICs) [3,4]. Ethiopia, the second most populous nation in Africa with a population exceeding 120 million, is projected to grow rapidly in the coming decades [5]. While infectious diseases remain dominant, the country is simultaneously facing a sharply increasing cancer burden [3].

According to GLOBOCAN 2022, lung cancer incidence and mortality rates in Ethiopia are 3.7 and 3.6 per 100,000 population, respectively [2]. However, these figures likely underestimate the true burden due to underdiagnosis [6] and the presence of only one population-based cancer registry for the capital city. Lung cancer arises from a combination of risk factors, including tobacco smoking, secondhand smoke (SHS), indoor and outdoor air pollution, occupational exposures, genetic predisposition, and chronic lung diseases such as tuberculosis, diet, and physical activity [2,7,8,9,10,11,12,13,14,15,16,17,18,19]. Their impact varies by place, demographics, genetics, and how these factors interact [1,20]. Although smoking is the dominant global driver of lung cancer, in Ethiopia, fewer than one-quarter of patients have ever smoked [21]. According to the Global Adult Tobacco Survey (2024), the prevalence of smoking in Ethiopia is 3.7% among adults, 7.2% among men, and 0.2% among women [22]. This suggests that additional exposures may play a major role in lung carcinogenesis in this setting. For instance, Ethiopia has one of the largest clean cooking access deficits globally, with solid-fuel use exceeding 94% [23]. The concentration of particulate matter (PM_2.5_) is five times higher than the acceptable threshold [24]. Ethiopia is also among the 30 nations with the highest tuberculosis burden [25], which may further contribute to the risk of lung cancer.

While research on the determinants of cancer has reached the molecular level in developed countries [20,26], in LMICs, including SSA, it remains in its infancy [20]. Moreover, the limited cancer research in SSA has primarily focused on breast and cervical cancers, leaving substantial knowledge gaps regarding lung cancer epidemiology, particularly risk factors beyond smoking. This knowledge gap is further compounded in Ethiopia by the existence of a single population-based cancer registry, which covers only the capital city, leaving the majority of the country’s 120 million population unrepresented.

Reduction in deaths from NCDs is on the global and national agenda [27]. In this regard, Ethiopia has developed national cancer control plans (2016 and 2025) [28,29] to reduce the cancer burden. These plans prioritize cancer prevention, early detection, diagnosis, and treatment. However, its progress remains limited, as witnessed by the rising burden of cancer, late presentation [6,21,30], and limited cancer care facilities [6,28]. Lung cancer is a preventable cause of death, but the effectiveness of prevention and early detection strategies depends on understanding locally relevant risk factors [4,20,31].

Guided by directed acyclic graphs (DAGs) [9,32], which are increasingly recommended in cancer epidemiology as a transparent and systematic approach for specifying hypothesized relationships and determining minimally sufficient covariate adjustment sets, the present study aims to identify lung cancer risk factors beyond smoking in Ethiopia.

## 2. Methods

### 2.1. Study Design, Setting, and Period

This multicenter matched case–control study was executed in Ethiopia from October 2023 to April 2025. This study was conducted as part of a multinational lung cancer diagnosis and control project in Ethiopia in collaboration with the Ministry of Health and seven partner organizations. It took place in three major teaching and referral hospitals: Tikur Anbessa Specialized Hospital (TASH) in Addis Ababa, Jimma University Medical Center (JUMC) in Jimma, and Haramaya University Hiwot Fana Comprehensive Specialized Hospital (HUHFCSH) in Harar, which are affiliated with Addis Ababa, Jimma, and Haramaya universities, respectively.

The three hospitals are the only hospitals providing cancer care services, including radiation, in Ethiopia. Notably, they are the only radiotherapy centers in Ethiopia [33]. TASH, the country’s largest referral hospital, was the sole provider of radiotherapy until April 2022, serving approximately 4.5 million people in Addis Ababa and receiving referrals from across the nation. JUMC, located 352 km southwest of Addis Ababa, is the oldest public hospital in the southwestern region, catering to a population of 15 million and housing Ethiopia’s second radiotherapy center. HUHFCSH, situated 510 km east of Addis Ababa, is the only comprehensive specialized hospital in eastern Ethiopia, serving over 5 million people and operating the country’s third radiotherapy center.

### 2.2. Sample Size Determination

The required sample size was calculated using the sample size formula for an individually matched case–control study design [34] with a 1:2 case-to-control ratio, assuming a 5% significance level (α) and statistical power (1-β) of 80%. Sample size estimates were derived for key exposures, including smoking [9,35], socioeconomic status [36,37], and chronic obstructive pulmonary disease [38,39], using exposure prevalence among controls and expected odds ratios. The largest estimated sample size for socioeconomic status was used as the minimum required sample. After accounting for a 10% potential non-response rate, the study enrolled 351 patients with lung cancer and 702 free-matched controls.

### 2.3. Selection of Cases and Controls

The cases were primary lung cancer patients with histopathological confirmation, recruited from the participating hospitals. Controls matched by sex, age (±5 years), and place of residence were selected from among patients who visited the same hospitals for acute health conditions unrelated to the exposures of interest (e.g., smoking, secondhand smoke, and occupational exposure). Controls were recruited from non-respiratory departments, such as ophthalmology and orthopedics, and had no history or clinical evidence of lung cancer. All potential controls were assessed by trained physicians with the consultation of pulmonologists and oncologists through clinical evaluation and review of medical records to ensure the absence of lung cancer.

### 2.4. Exposure Assessment

Data were collected using a validated structured questionnaires designed based on the DAG framework [32]. The questionnaire consisted of six sections covering sociodemographic and economic characteristics, occupational and environmental exposures, behavioral factors, and medical history. Sociodemographic and economic information included age, sex, place of residence, educational status, and household assets. Household assets were used to construct a wealth index using principal component analysis (PCA). We used a classification of occupations created by Ahrens and Merletti [10], based on occupational categories (ISCO-68) and industrial sectors, to capture occupational exposures to lung carcinogens (ISIC Rev.2). Due to small numbers in specific list A and list B occupations, occupational exposure was dichotomized as ever or never having worked in a list A or B job.

To assess household solid-fuel use, participants were asked about the types of fuels primarily used for cooking or heating. Although additional information on cooking location, ventilation, duration of use, and number of meals per day was collected, following Demographic Health Survey (DHS) guidelines, exposure classification for this study was based on the primary fuel type [23] to simplify interpretation, reduce misclassification related to recall, and ensure comparability with prior studies. Participants were classified as solid-fuel users if their primary fuel was a solid fuel and as non-solid-fuel users if their primary fuel was a clean fuel.

Smoking exposure was categorized using standard epidemiologic criteria—ever-smokers (≥100 cigarettes) and never-smokers (<100 cigarettes) [11]. Secondhand smoke exposure was assessed separately for household and workplace settings. Participants were classified as ever exposed if they reported regular exposure to SHS (living with or working near smokers) for ≥6 months and as never exposed otherwise.

Long-term physical activity during the 10 years preceding diagnosis [15] was assessed using a short-form lifetime physical activity questionnaire adapted from the validated lifetime physical activity questionnaire (LTPAQ). Participants reported typical activity in four domains (occupational, household, transport, and recreational), including months per year, hours per week during active months, and years engaged in the activity during the 10-year period. Metabolic Equivalent of Task (MET) values were set at 3.3 for walking, 4.0 for moderate, and 8.0 for vigorous activities based on WHO standards. Total MET-minutes per week were calculated by averaging weekly MET-minutes over the 10-year window. Physical activity levels were categorized according to WHO recommendations. We classified participants as sufficiently active if they achieved 600 MET-minutes per week or more, whereas those below this were considered insufficiently active [40].

Dietary patterns were assessed by a food frequency questionnaire (FFQ). Latent Class Analysis (LCA) was applied to identify dietary patterns based on participants’ consumption of three food groups. Model selection was guided by Bayesian Information Criterion (BIC), entropy, interpretability, and class separation, and a three-class solution was selected. The classes were labeled as meat-based, cereal- and vegetable-based, and processed food-based, based on the food groups with the highest probabilities within each class. Participants were assigned to classes based on the highest posterior probability. These dietary classes were used as categorical independent variables in regression analyses to evaluate associations with lung cancer risk.

A prior history of pulmonary tuberculosis (TB) was ascertained by self-report physician diagnosis and, where available, by reviewing clinical records. To reduce misclassification of early lung cancer as TB and the potential for reverse causation, the primary exposure definition classified participants as TB-exposed only if TB was diagnosed ≥2 years before the lung cancer diagnosis (or interview date for controls) [19,41]. The history of COPD was assessed using medical records and self-reported physician diagnosis. To classify COPD relative to lung cancer, pre-existing COPD was defined as a diagnosis recorded at least 1 year prior to the lung cancer diagnosis.

Family history of cancer was assessed by asking participants whether any biological relative had been diagnosed with lung cancer or any cancer, followed by the relationship of the affected relative [42]. Due to small numbers reporting lung cancer specifically, a composite variable indicating any family history of cancer was created for analysis. Tablet-based in-person interviews were conducted using Kobo Toolbox v2022.2.3. Trained health professionals with prior data collection experience, who were blinded to case/control status, collected the data. Data collection was supervised by MSc-level public health professionals through regular monitoring and review to ensure adherence to the study protocol. Standardized procedures were applied across all sites.

### 2.5. Data Processing and Analysis

**Figure 1:**
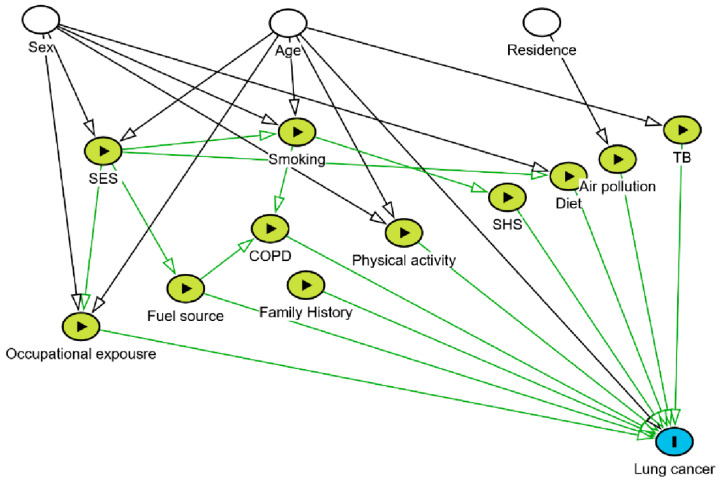
DAG on the potential risk factors of lung cancer in Ethiopia. SES: socioeconomic status, COPD: chronic obstructive pulmonary disease, SHS: secondhand smoke, and TB: tuberculosis.

Participant characteristics were summarized using frequency and percentages. A DAG was developed to guide the hypothesized relationships between lung cancer and its potential risk factors. Nodes represent variables and directed edges indicate presumed effects, with no feedback loops [32]. The DAG drew on evidence from the IARC [43], published literature [2,7,8,9,10,11,12,13,14,15,16,17,18,19], and experts’ input from the Ethiopian context. It guided variable selection for multivariable hierarchical conditional logistic regression analyses through identifying the minimally sufficient adjustment sets for each exposure and informing model specification (Figure 1).

Bivariable conditional logistic regressions were estimated to assess the crude odds ratio between lung cancer and each potential risk factor. All variables specified in the directed acyclic graph (DAG), constructed a priori based on existing evidence and biological plausibility, were included in the final conditional hierarchical multivariable logistic regression model to ensure appropriate adjustment for confounding. Variables were entered sequentially in four conceptually defined blocks aligned with the ordering in the DAG: (1) demographic and socioeconomic characteristics (education and wealth); (2) environmental and occupational exposures (biomass fuel and occupational exposure); (3) behavioral factors (diet, physical activity, smoking, and secondhand smoke) and (4) medical history (tuberculosis, chronic obstructive pulmonary diseases and family history of cancer). Multicollinearity was checked, and model fit was evaluated with log-likelihood (LL), likelihood ratio tests, AIC, and BIC.

A subgroup analysis was conducted to assess whether the associations between lung cancer and its risk factors differed by sex. We also attempted a subgroup analysis by smoking status; however, the non-smoker subgroup model converged, whereas the smoker subgroup did not converge due to the small number of participants reporting smoking. The robustness of our main multivariable hierarchical conditional logistic regression model was assessed by multiple sensitivity analyses. We fitted nine additional leave-one-out models, each excluding one DAG-informed variable at a time (i.e., smoking, SHS, cooking fuel, occupational exposure, COPD, TB, diet, and physical activity). To further validate the conditional logistic regression findings, we fitted a traditional (unconditional) logistic regression model using the same set of covariates to assess consistency in the direction and magnitude of the associations. In addition, because some variables—particularly in the sex-stratified models—had sparse cells or low event counts, we conducted sensitivity analyses using Firth penalized logistic regression to address potential small-sample and separation bias.

Analyses were done using Stata version 19.5 (StataCorp LLC, College Station, TX, USA) and the DAG drawings using the R package “dagitty” [32]. All subgroup and sensitivity analysis results are shared as a supplementary file. This study followed the STROBE reporting guidelines and adhered to best practices for inference reporting using DAGs.

## 3. Results

### 3.1. Characteristics of the Participants

A total of 1053 participants, including 351 lung cancer cases and 702 controls matched on age, sex, and residence, were included in the study. The bulk of the sample was aged 45 years or more, and males slightly outnumbered females. Educational levels were mixed, with about two-fifths having secondary education or higher and the rest split between primary education and no formal education. Wealth was evenly distributed across categories. Over half reported using solid fuels for cooking. Occupational exposure, active smoking, and secondhand smoke were relatively uncommon. More than half had insufficient physical activity, and fewer than half followed a plant-based diet. Only a small proportion reported tuberculosis, COPD, or a family history of cancer (Table 1).

### 3.2. Predictors of Lung Cancer

All variables included in the analysis were selected using a DAG reflecting Ethiopia’s lung cancer risk landscape beyond smoking. The DAG guided the inclusion of socioeconomic factors, household fuel exposure, occupational hazards, lifestyle behaviors, comorbidities, and family history to ensure a conceptually motivated rather than a purely data-driven model. Cross-tabulation results showed notable differences between the two groups. The bivariable conditional logistic regression showed notable crude associations with lung cancer. Compared with controls, lung cancer cases had lower educational status and were more likely to be in the low-wealth category. Solid-fuel use for cooking was substantially more common among cases. Occupational exposure, active smoking, and secondhand smoke were also more frequent among cases. Insufficient physical activity and plant-based diets were less common among cases. A history of tuberculosis was more prevalent among cases, whereas COPD and family history of cancer showed no significant differences. These crude associations are summarized in Table 2.

Four hierarchical conditional logistic regression models (M1–M4) were fitted to identify lung cancer risk factors. Model 1 (M1) included only the main exposure variables, Model 2 (M2) added sociodemographic factors such as education and wealth, Model 3 (M3) further incorporated behavioral and environmental variables, including smoking, cooking fuel, and occupational exposures, and Model 4 (M4), the final model, additionally adjusted for clinical and family history variables. Each successive model significantly improved fit over the previous one, as demonstrated by likelihood-ratio tests (M1 vs. M2: χ^2^ (2) = 28.07, *p* < 0.001; M2 vs. M3: χ^2^ (5) = 110.87, *p* < 0.001; M3 vs. M4: χ^2^ (3) = 21.67, *p* < 0.001). The final model (M4) had the best overall fit (LL = −273.61, df = 14, AIC = 575.23, and BIC = 644.66), with progressively lower AIC and BIC values across the models, confirming improved explanatory capacity with the addition of sociodemographic, behavioral, environmental, and clinical predictors.

Based on the final multivariable hierarchical conditional logistic regression, higher education remained protective: primary education was associated with lower odds of lung cancer [(AOR = 0.54, 95% CI 0.36, 0.80; *p* = 0.002) and secondary or above also showed reduced odds (AOR = 0.49, 95% CI 0.30, 0.81; *p* = 0.006) compared with illiterate participants. Low household wealth was strongly associated with increased odds (AOR = 2.27, 95% CI 1.43, 3.58; *p* < 0.001). Household use of solid fuel remained an independent risk factor (AOR = 1.80, 95% CI 1.26–2.52; *p* = 0.001), as did occupational exposure to dust/chemicals (AOR = 1.90, 95% CI 1.05–3.40; *p* = 0.032). Smoking showed a large effect (AOR = 3.90, 95% CI 2.21, 6.87; *p* < 0.001), and secondhand smoke exposure was associated with higher odds (AOR = 2.70, 95% CI 1.50–4.84; *p* = 0.001). Sufficient physical activity was protective (AOR = 0.32, 95% CI 0.23, 0.45; *p* < 0.001), and a processed-food-based dietary pattern was associated with increased odds (AOR = 1.70, 95% CI 1.07, 2.68; *p* = 0.023). A history of tuberculosis was strongly associated with lung cancer (AOR = 3.21, 95% CI 1.71, 6.01; *p* < 0.001), whereas COPD showed a non-significant association (AOR = 1.99, 95% CI 0.89, 4.43; *p* = 0.091). A family history of cancer was linked with higher odds (AOR = 2.10, 95% CI 1.01, 4.37; *p* = 0.046) (Table 3).

The subgroup analysis by sex showed consistent associations across males and females, including low education, low income, insufficient physical activity, and a history of tuberculosis. However, the subgroup analysis suggests that smoking, occupational hazards, meat-based dietary pattern, and family history have stronger effects among males, whereas secondhand smoke, solid fuels, and processed food dietary pattern show clearer associations among females (Table 4). Subgroup analysis, including cross-tabulation, COR, AOR with 95% CI, and *p*-values, is provided in Supplementary Tables S1 and S2, and the subgroup analysis for non-smokers is provided in Supplementary Table S3.

To evaluate the robustness of the multivariable hierarchical conditional logistic regression model and assess whether potential mediators identified through the DAG could unduly influence model stability, we fitted nine additional leave-one-out models, each excluding one DAG-informed variable at a time (i.e., smoking, SHS, cooking fuel, occupational exposure, COPD, TB, diet, and physical activity). Removing one exposure at a time did not substantially change the magnitude or significance of most key predictors, indicating the findings are robust (Supplementary Table S4). To further validate the conditional logistic regression findings, we fitted a traditional (unconditional) logistic regression model using the same set of covariates to assess consistency in the direction and magnitude of the associations (Supplementary Table S5). In addition, because some variables—particularly in the sex-stratified models—had sparse cells or low event counts, we conducted sensitivity analyses using Firth penalized logistic regression to address potential small-sample and separation bias (Supplementary Table S6). The sensitivity analysis largely confirmed the direction and magnitude of associations observed in the primary models. Minor attenuation observed for occupational exposure and family history suggests some sensitivity to model specification but does not materially alter the overall conclusions.

## 4. Discussion

While smoking is a predominant driver of lung cancer cases worldwide [2,20], in Ethiopia, fewer than a quarter of patients have ever smoked, pointing to a unique pattern of local risk factors [21]. Evidence to inform policy regarding local risk factors in resource-limited settings is lacking [1,20]. Guided by a DAG [32], this study identified several factors independently associated with lung cancer beyond smoking, including low education and wealth, solid-fuel use, occupational exposures, insufficient physical activity, diet, SHS, prior tuberculosis history, and a family history of cancer. A subgroup analysis by sex showed consistent associations across males and females. However, differences in exposure distribution are explained by sex-specific patterns: smoking, occupational exposure, meat-based diet, and family history are more common in males, whereas secondhand smoke, solid fuels, and processed food dietary patterns are more prevalent in females.

The findings from this study reinforce the impact of socioeconomic status on lung cancer risk [37,44]. Low levels of education and wealth were associated with a higher risk of lung cancer. Similarly, evidence from a Mendelian randomization study also supported low income and education as being related to the risk of lung cancer [44]. The precise mechanisms by which socioeconomic status influences lung cancer risk remain under investigation [37]. However, most studies agree that lung cancer is affected by lifestyle factors, which in turn are influenced by socioeconomic status [37,44]. In resource-limited settings like Ethiopia, socioeconomic status plays a central role in shaping lung cancer. For instance, the use of clean fuel can depend on SES, and those with a lower SES may engage in at-risk work [1,20]. Hence, targeting lower-SES groups can potentially improve the outcomes of lung cancer prevention and control interventions.

Smoking and SHS are established risk factors for lung cancer [1,12,13,16,20]. Unsurprisingly, both smoking and SHS are linked with lung cancer risk in our study. Our subgroup analysis further shows that smoking disproportionately affects males, whereas SHS affects females. These findings closely mirror the national exposure profile reported by the 2024 Global Adult Tobacco Survey (GATS) in Ethiopia, which documented substantially higher smoking prevalence among males (7.2%) compared with females (0.2%), alongside a relatively high prevalence of SHS exposure (11%) [22]. In LMICs where resources are limited, prevention work should be given the highest priority [28]. Smoking is one of the preventable causes but the leading cause of lung cancer and other NCDs [2,14,20]. Hence, the reduction in smoking has multiple benefits in the reduction in NCDs. In this regard, Ethiopia has developed a cancer control plan [28] that focuses on reducing smoking and introducing tobacco control policies to prevent SHS [45]. Full implementation of these plans and strengthening such public health measures could meaningfully reduce the burden not only of lung cancer but also of most NCDs.

Household solid-fuel use is another crucial factor associated with the risk of lung cancer, which disproportionately affects females. Solid-fuel use is the primary source of energy in LMICs [1,20]. Globally, Ethiopia remains a country with one of the largest clean cooking access deficits, with approximately 94% of the population relying on solid fuels for cooking and heating [23]. Household air pollution from wood, charcoal, and straw is reported as carcinogenic among never-smoker women [7,11]. According to the WHO report, 11% of lung cancer deaths are attributable to solid-fuel use [46]. Women and children are at high risk for exposure to solid fuel in our context [47]. This may explain the high burden of lung cancer among non-smoking women in Ethiopia. Given this, lung cancer prevention and control targeting solid-fuel use could be beneficial in resource-limited settings. At the same time, targeting solid-fuel users as a high-risk group for early detection could be useful among never-smoker women. Expanding clean energy use as a country is also a long-term and sustainable solution.

In our study, prior tuberculosis emerged as a significant risk factor of lung cancer, consistent with previous studies from many LMICs [19,48]. Pooled analysis from 32 articles reported a relative risk of 2.2 [48]. Although few localized studies in SSA have directly examined the link between prior TB and lung cancer, evidence from post-tuberculosis lung disease research [49] shows that nearly half of TB survivors develop chronic pulmonary sequelae, including airflow limitation and fibrosis, which may increase subsequent lung cancer risk. The relationship between lung cancer and tuberculosis is particularly relevant in our context, because Ethiopia is among the 30 high-TB-burden countries [25]. TB can cause lung cancer tissue destruction, fibrosis, and DNA damage, which makes this relationship biologically plausible [48]. However, the reverse causation may also be true [41], as early-stage lung cancer may predispose to TB or be misdiagnosed due to overlapping symptoms, presenting substantial diagnostic challenges in resource-limited settings [6]. Given the link between TB and lung cancer, existing TB programs offer an opportunity to integrate lung cancer screening and early detection [19,48,50]. TB-established infrastructure for imaging, sputum testing, and follow-up can be leveraged for dual screening, prioritizing high-risk patients such as those with recurrent TB, persistent lesions, or post-TB lung disease. Training TB program staff to recognize red flags for malignancy can further reduce diagnostic gaps. Such integration also supports broader efforts to strengthen non-communicable disease services within infectious disease platforms, promoting more comprehensive, patient-centered care. Efforts to end TB can also help reduce the lung cancer burden.

Occupational exposure is common in industries such as mining, construction, and transport; asbestos is the most well-documented occupational carcinogen [1,8,9,10,20]. The IARC classifies carcinogenic agents into a confirmed list A and a suggestive list B based on available evidence. In our study, the number of participants who reported working on both list A and B was small. However, our study findings revealed a non-negligible risk of occupational exposure, particularly among men, given the low occupational health standards in LMICs. Further research with objective exposure measurements could help quantify these risks.

In this study, behavioral factors, particularly low physical activity and unhealthy dietary patterns, were associated with lung cancer risk [1,15,51,52]. This finding aligns with global evidence linking lifestyle factors to NCDs. SSA is currently undergoing rapid demographic and epidemiological transitions, characterized by urbanization, shifting physical activity, and sociocultural dietary patterns [3]. Recent local evidence showed both an increase in physical inactivity [40] and changes in dietary patterns [53] in Ethiopia. Taken together, it implies the need to integrate lifestyle-based prevention strategies into border lung cancer prevention strategies. However, due to the long-term assessment of physical activity and dietary patterns, the interpretation of this finding should consider the potential for reverse causation and recall bias. Hence, further prospective research is warranted.

Having a family history of cancer was linked to higher odds of developing lung cancer, consistent with evidence from previous research [17,18]. A study showed the relative risk for lung cancer from an additional first-degree family history ranging from 2.57 to 4.24 [42]. Although family history is a non-modifiable risk factor, it remains clinically relevant because it helps identify individuals at elevated baseline risk [17,18]. This is particularly important in Ethiopia because late-stage presentation is common, and the majority of lung cancer patients are never-smokers [21]. Inclusion of family history as criteria for target screening showed promising results in a study conducted in Twain among non-smokers [54]. However, interpretation of our finding should be considered with caution since the number of participants with a family history of cancer or lung cancer was small.

The growing burden of NCDs in LMICs like Ethiopia is undeniable [3]. The relation between tuberculosis and lung cancer exemplifies the need for integrated control strategies for both communicable and NCDs. Our findings imply the relevance of local risk factors beyond smoking on lung cancer risk in resource-limited settings. Prevention and early detection strategies need to target risk factors beyond smoking, considering sex-specific patterns. Because risk factors are shared across NCDs, coordinated interventions can produce wide-reaching health benefits. The findings from this study could inform a context-based prevention and control, detection, diagnosis, and treatment guideline for lung cancer.

## 5. Strengths and Limitations of the Study

This study has several strengths. To our knowledge, this is the first multicenter matched case–control study assessing lung cancer risk factors using a DAG in SSA. Notably, even at the global level, research on risk factors among non-smokers and younger patients with lung cancer remains limited. Our study helps bridge this gap, as around three-fourths of lung cancer patients in this study were non-smokers and a significant proportion were younger, providing valuable insights into this understudied group. Several methodological strengths enhance the quality of the study, including the use of a multicenter matched case–control design with a relatively large sample size, assessment of multiple exposures, application of a multivariable hierarchical model, and the conduct of subgroup and sensitivity analysis. This study also has limitations. First, recall bias is possible because exposure information was collected retrospectively using self-reported data. Second, the use of hospital-based controls may limit the representativeness of the source population, potentially introducing selection bias, as exposures could be associated with other hospital-attended conditions. Additionally, although the three participating hospitals are major referral centers serving diverse populations across Ethiopia, access to these facilities may be easier for urban residents, while rural populations may face geographic and structural barriers. Residual confounding is possible due to the absence of objective measurements of air pollution and radon exposure, despite adjustments for residence. Smoking history was collected but not fully analyzed because of the small number of smokers, limiting dose–response assessment. Future studies could consider prospective cohort designs, integrating objective air pollution monitoring, biomarker validation, standardized clinical assessments, and longitudinal follow-up of post-TB patients.

## 6. Conclusions

In Ethiopia, lung cancer appears to be associated with a combination of environmental, occupational, behavioral, and medical factors beyond smoking. While most risk factors affect both sexes, sex-specific exposure patterns explain differences in risk. Our findings highlight the need for multi-pronged, gender-sensitive public health interventions addressing smoking, secondhand smoke, biomass fuel use, occupational exposure, lifestyle behaviors, and tuberculosis. Targeting these locally relevant risk factors is needed for effective lung cancer prevention and early detection in Ethiopia and similar resource-limited settings.

## Figures and Tables

**Table 1:** Characteristics of the study participants (cases and controls combined).

Variables	Category	Frequency (n = 1053)	Percent (%)
Age	Less than 45	296	28.11
	45 and above	757	71.89
Sex	Female	507	48.15
	Male	546	51.85
Education	Primary (1–8)	343	32.57
	Secondary & above	409	38.85
	No formal education	301	28.58
Wealth	High	323	30.67
	Low	360	34.19
	Medium	370	35.14
Household solid-fuel use	Solid-fuel user	594	58.59
	Non-solid-fuel user	459	41.41
Occupational exposure	Ever	95	9.10
	Never	958	90.9
Smoking	Ever	97	9.21
	Never	956	90.79
Secondhand smoke	Ever	75	7.12
	Never	978	92.88
Physical activity	Sufficient	470	44.63
	Insufficient	583	55.37
Dietary pattern	Cereal- & plant-based	501	47.58
	Meat-based	410	38.93
	Processed food-based	142	13.49
Tuberculosis	Yes	63	6.00
	No	990	94.0
COPD ^a^	Yes	44	4.18
	No	1009	95.82
Family history of cancer	Yes	52	4.90
	No	1001	95.1

List A: occupations with well-established lung carcinogen exposure; list B: occupations with suspected lung carcinogen exposure (Ahrens & Merletti); ^a^ chronic obstructive pulmonary diseases.

**Table 2:** Crude conditional logistic regression results for lung cancer risk factors.

Variables	Cases	Controls	COR with 95% CI
Education			
Secondary & above	132 (37.6%)	277 (39.5%)	0.51 (0.37–0.71) ***
Primary (1–8)	67 (19.1%)	234 (33.3%)	0.29 (0.20–0.43) ***
No formal education	152 (43.3%)	191 (27.2%)	reference
Wealth			
Low	167 (47.6%)	193 (27.5%)	3.00 (2.12–4.25) ***
Medium	110 (31.3%)	260 (37.0%)	1.49 (1.04–2.15) *
High	74 (21.1%)	249 (35.5%)	reference
Household solid-fuel use			
Solid-fuel user	246 (70.1%)	348 (49.6%)	2.58 (1.93–3.45) ***
Non-solid-fuel user	105 (29.9%)	354 (50.4%)	reference
Occupational exposure			
Ever	39 (11.1%)	54 (7.7%)	1.61 (1.01 –2.59) *
Never	312 (88.9%)	648 (92.3%)	reference
Smoking			
Ever	60 (17.1%)	37 (5.3%)	4.56 (2.78–7.50) ***
Never	291 (82.9%)	665 (94.7%)	reference
Secondhand smoke			
Ever	35 (10.0%)	40 (5.7%)	1.80 (1.13–2.86) *
Never	316 (90.0%)	662 (94.3%)	reference
Physical activity			
Sufficient	263 (74.9%)	320 (45.6%)	0.26 (0.19–0.35) ***
Insufficient	88 (25.1%)	382 (54.4%)	reference
Dietary pattern			
Meat-based	62 (17.7%)	80 (11.3%)	1.35 (1.02–1.79) *
Processed food-based	145 (41.3%)	265 (37.8%)	1.83 (1.27–2.64) **
Cereal- & plant-based	144 (41.0%)	357 (50.9%)	reference
Tuberculosis			
Yes	38 (10.8%)	25 (3.6%)	3.19 (1.89–5.36) ***
No	313 (89.2%)	677 (96.4%)	reference
COPD ^a^			
Yes	16 (4.60%)	28 (3.9%)	1.14 (0.62–2.11)
No	335 (95.4%)	674 (96.1%)	reference
Family history of cancer			
Yes	23 (6.5%)	29 (4.1%)	1.70 (0.95–3.06)
No	328 (93.5%)	673 (95.9%)	reference

* *p* < 0.05; ** *p* < 0.01; *** *p* < 0.001; ^a^ COPD: chronic obstructive pulmonary diseases.

**Table 3:** Multivariable hierarchical conditional logistic regression results for lung cancer risk factors.

	AOR with 95% CI ^a^			
Variables	Model 1	Model 2	Model 3	Model 4
Education				
Primary (1–8)	0.56 (0.39–0.78)	0.59 (0.41–0.83)	0.49 (0.33–0.73)	0.54 (0.36–0.80) **
Secondary & above	0.43 (0.28–0.66)	0.54 (0.34–0.84)	0.52 (0.31–0.84)	0.49 (0.29–0.81) **
No formal education	reference	reference	reference	reference
Wealth				
Low	2.29 (1.55–3.38)	2.19 (1.46–3.28)	2.21 (1.41–3.44)	2.27 (1.43–3.58) ***
Medium	1.33 (0.90–1.97)	1.29 (0.86–1.94)	1.09 (0.70–1.72)	1.10 (0.70–1.75)
High	reference	reference	reference	reference
Household solid-fuel use				
Solid-fuel user		2.05 (1.49–2.80)	1.78 (1.27–2.50)	1.78 (1.26–2.52) **
Non-solid-fuel user		reference	reference	reference
Occupational exposure				
Ever		2.14 (1.27–3.60)	1.99 (1.12–3.56)	1.89 (1.05–3.40) *
Never		reference	reference	reference
Smoking				
Ever			4.17 (2.40–7.23)	3.90 (2.21–6.87) ***
Never			reference	reference
Secondhand smoke				
Ever			2.80 (1.59–4.92)	2.70 (1.50–4.84) **
Never			reference	reference
Physical activity				
Sufficient			0.33 (0.23–0.45)	0.32 (0.23–0.45) ***
Insufficient			reference	reference
Dietary pattern				
Meat-based			1.23 (0.88–1.71)	1.35 (0.95–1.89)
Processed food-based			1.57 (1.01–2.46)	1.70 (1.07–2.68) *
Cereal- & plant-based			reference	reference
Tuberculosis				
Yes				3.21 (1.71–6.01) ***
No				reference
COPD ^b^				
Yes				1.99 (0.89–4.43)
No				reference
Family History of Cancer				
Yes				2.10 (1.01–4.37) *
No				reference

^a^ Adjusted odds ratio with 95% confidence interval; * *p* < 0.05; ** *p* < 0.01; *** *p* < 0.001; ^b^ chronic obstructive pulmonary diseases.

**Table 4:** Sex-specific multivariable conditional logistic regression models assessing risk factors for lung cancer.

	Male		Female	
Variables	COR (95% CI) ^a^	AOR (95% CI) ^b^	COR (95% CI)	AOR (95% CI)
Education				
Primary	0.56 (0.36–0.89)	0.63 (0.35–1.11) *	0.48 (0.30–0.76)	0.55 (0.30–1.02) *
Secondary & above	0.36 (0.22–0.60)	0.53 (0.28–1.03) *	0.22 (0.12–0.41)	0.59 (0.25–1.37)
No formal education	reference	reference	reference	reference
Wealth				
Low	2.54 (1.57–4.11)	2.47 (1.25–4.89) **	3.37 (2.02–5.61)	2.13 (1.10–4.22) *
Medium	1.66 (1.04–2.67)	1.44 (0.76–2.71)	1.31 (0.73–2.32)	0.90 (0.43–1.86)
High	reference	reference	reference	reference
Household solid-fuel use				
Solid-fuel user	1.83 (1.25–2.68)	1.46 (0.91–2.34)	4.00 (2.52–6.34)	2.67 (1.50–4.73) **
Non-solid-fuel user	reference	reference	reference	reference
Occupational Exposure				
Ever	2.32 (1.22–4.43)	4.01 (1.71–9.42) **	1.05 (0.50–2.17)	0.75 (0.29–1.90)
Never	reference	reference	reference	reference
Smoking				
Ever	5.48 (3.05–9.86)	4.70 (2.36–9.36) ***	2.60 (0.99–6.86)	2.52 (0.84–7.62)
Never	reference	reference	reference	reference
Secondhand smoke				
Ever	1.47 (0.79–2.74)	2.12 (0.88–5.08)	2.34 (1.15–4.76)	3.58 (1.48–8.70) **
Never	reference	reference	reference	reference
Physical activity				
Sufficient	0.29 (0.19–0.44)	0.31 (0.19–0.50) ***	0.23 (0.15–0.36)	0.30 (0.18–0.50) ***
Insufficient	reference	reference	reference	reference
Dietary pattern				
Meat based	1.28 (0.87–1.88)	1.63 (1.00–2.65) *	1.44 (0.96–2.17)	1.20 (0.72–2.01)
Processed food-based	1.65 (0.98–2.78)	1.52 (0.77–2.99)	2.02 (1.20–3.40)	2.11 (1.09–4.08) *
Cereals & plant-based	reference	reference	reference	reference
Tuberculosis				
Yes	3.45 (1.70–7.00)	4.36 (1.66–11.5) **	2.90 (1.35–6.27)	2.80 (1.10–7.11) *
No	reference	reference	reference	reference
COPD ^c^				
Yes	1.06 (0.47–2.38)	2.67 (0.88–8.11)	1.27 (0.49–3.28)	1.52 (0.44–5.27)
No	reference	reference	reference	reference
Family History of Cancer				
Yes	5.00 (1.94–12.8)	4.46 (1.49–13.4) **	0.62 (0.24–1.58)	0.93 (0.28–3.01)
No	reference	reference	reference	reference

^a^ Crude odds ratio with 95% confidence interval. ^b^ Adjusted odds ratio with 95% confidence interval; * *p* < 0.05; ** *p* < 0.01; *** *p* < 0.001; ^c^ COPD: chronic obstructive pulmonary disease.

## Data Availability

All relevant data are within the paper and its supporting information files. Upon reasonable request, the corresponding author will provide the raw data used in the study.

## Supplementary Materials

The following supporting information can be downloaded at: https://www.mdpi.com/article/10.3390/cancers18060914/s1, Supplementary Table S1: Sex-specific multivariable conditional logistic regression model for lung cancer risk factors among males; Supplementary Table S2: Sex-specific multivariable conditional logistic regression model for lung cancer risk factors among females; Supplementary Table S3: Multivariable conditional logistic regression model for lung cancer risk factors among never-smokers; Supplementary Table S4: Leave-one-out multivariable conditional logistic regression model for lung cancer risk factors beyond smoking in Ethiopia; Supplementary Table S5: Multivariable logistic regression model for lung cancer risk factors beyond smoking in Ethiopia; Supplementary Table S6: Multivariable Firth logistic regression model for lung cancer risk factors beyond smoking in Ethiopia.

## Abbreviations

The following abbreviations are used in this manuscript:

DHS	Demographic Health Survey
GLOBOCAN	Global Cancer Observatory
HUHFCSH	Hiwot Fana Comprehensive Specialized Hospital
ICH-GCP	International Council for Harmonisation—Good Clinical Practice
JUMC	Jimma University Medical Center
LMICs	Low- and Middle-Income Countries
LTPAQ	Lifetime Physical Activity Questionnaire
MET	Metabolic Equivalent of Task
NCDs	Non-Communicable Diseases
SDG	Sustainable Development Goal
SSA	Sub-Saharan Africa
TASH	Tikur Anbessa Specialized Hospital
TB	Tuberculosis
WHO	World Health Organization
