# Supplementary material for: Lung Cancer Risk Factors Beyond Smoking in Ethiopia: A Multicenter Matched Case–Control Study
Source: Cancers (Basel). 2026 Mar 12;18(6):914. doi: 10.3390/cancers18060914 (PMC13024100; doi:10.3390/cancers18060914)
Supplement: Supplementary file 1 [file cancers-18-00914-s001.zip › cancers-4148749-supplementary.pdf]

Contents Page

Supplementary Table S1. Sex-specific multivariable conditional logistic regression model for Lung cancer risk factors among males 2

Supplementary Table S2. Sex-specific multivariable conditional logistic regression model for lung cancer risk factors among females 4

Supplementary Table S3. Multivariable conditional logistic regression model for lung cancer risk factors among never-smokers 6

Supplementary Table S4.1: Leave-one-out multivariable conditional logistic regression model for lung cancer risk factors beyond smoking in Ethiopia 7

Supplementary Table S4.2: Leave-one-out multivariable conditional logistic regression model for lung cancer risk factors beyond smoking in Ethiopia 9

Supplementary Table S5: Multivariable logistic regression model for lung cancer risk factors beyond smoking in Ethiopia 11

Supplementary Table S6. Multivariable firth logistic regression model for lung cancer risk factors beyond smoking in Ethiopia 13

Supplementary Table S1. Sex-specific multivariable conditional logistic regression model for Lung cancer risk factors among males

**Variables**	**Cases**	**Controls**	**COR (95% CI)**	**AOR (95% CI)**
Education				
Primary (1-8)	73 (40.1%)	147 (40.4%)	0.56 (0.36,0.89)	0.63 (0.35–1.11)
Secondary & above	45 (24.7%)	139 (38.2%)	0.36 (0.22,0.60)	0.53 (0.28–1.03)
No formal education	64 (35.2%)	78 (21.4%)	reference	reference
Wealth				
Low	66 (36.2%)	84 (23.1%)	2.54 (1.57,4.11)	2.47 (1.25, 4.89) **
Medium	68 (37.4%)	134 (36.8%)	1.66 (1.04,2.67)	1.44 (0.76, 2.71)
High	48 (26.4%)	146 (40.1%)	reference	reference
Household solid fuel use				
Solid-fuel user	114(62.6%)	178 (48.9%)	1.83(1.25,2.68)	1.46 (0.91,2.34)
Nonsolid-fuel user	68(37.4%)	186(51.1%)	reference	reference
Occupational Exposure				
Ever	28 (15.4%)	32 (9.00%)	2.32 (1.22,4.43)	4.01 (1.71, 9.42) **
Never	154 (84.6%)	332 (91.0%)	reference	reference
Smoking				
Ever	49 (26.9%)	27 (7.40%)	5.48 (3.05,9.86)	4.7 (2.36, 9.36) ***
Never	133(73.1%)	337 (92.6%)	reference	reference
Secondhand smoke				
Ever	18 (9.90%)	25 (6.90%)	1.47(0.79, 2.74)	2.12 (0.88, 5.08)
Never	164(93.1%)	339 (93.1%)	reference	reference
Physical activity				
Sufficient	40 (22.0%)	180 (49.5%)	0.29 (0.19,0.44)	0.31 (0.19,0.50) ***
Insufficient	142(78.0%)	184 (50.5%)	reference	reference
Dietary pattern				
Meat-based	74 (40.7%)	137 (37.7%)	1.28 (0.87,1.88)	1.63 (1.00, 2.65) *
Processed food based	30 (16.5%)	42 (11.5%)	1.65 (0.98,2.78)	1.52 (0.77,2.99)
Cereals & Plant based	78 (42.9%)	185 (50.8%)	reference	reference

Tuberculosis				
Yes	22 (12.1%)	14 (4.00%)	3.45 (1.70,7.00)	4.36 (1.66,11.47) **
No	160 (87.9%)	350 (96.0%)	reference	reference
COPD				
Yes	9 (4.90%)	17 (5.00%)	1.06 (0.47,2.38)	2.67 (0.88,8.11)
No	173(95.1%)	347(95.0%)	reference	reference
Family History of cancer				
Yes	17 (9.30%)	10 (2.75%)	5.00 (1.94,12.8)	4.46 (1.49,13.36) **
No	165(90.7%)	354 (97.3%)	reference	reference

Note: COPD: chronic obstructive pulmonary diseases, * $p < 0.05$; ** $p < 0.01$; *** $p < 0.001$

Supplementary Table S2. Sex-specific multivariable conditional logistic regression model for lung cancer risk factors among females

**Variables**	**Cases**	**Controls**	**COR (95% CI)**	**AOR (95% CI)**
Education				
Primary (1-8)	59 (34.9%)	130 (38.5%)	0.48 (0.30, 0.76)	0.55 (0.30,1.02)
Secondary & above	22 (13.1%)	95 (28.1%)	0.22 (0.12, 0.41)	0.59 (0.25,1.37)
No formal education	88 (52.0%)	113 (33.4%)	reference	reference
Wealth				
Low	101(59.7%)	109 (32.2%)	3.37 (2.02, 5.61)	2.13 (1.10,4.22) *
Medium	42 (37.3%)	126 (37.3%)	1.31 (0.73, 2.32)	0.90 (0.43,1.86)
High	26 (15.0%)	103 (30.5%)	reference	reference
Household solid fuel use				
Solid-fuel user	132(78.1%)	170 (50.3%)	4.00 (2.52, 6.34)	2.67 (1.50,4.73) **
Nonsolid-fuel user	37(21.9%)	168 (49.7%)	reference	reference
Occupational Exposure				
Ever	12 (7.1%)	23 (6.8%)	1.05 (0.50, 2.17)	0.75 (0.29,1.90)
Never	157(92.9%)	315(93.2%)	reference	reference
Smoking				
Ever	11 (6.50%)	10 (2.90%)	2.60 (0.99, 6.86)	2.52 (0.84,7.62)
Never	158(93.5%)	328 (97.1%)	reference	reference
Secondhand smoke				
Ever	17 (10.1%)	15 (4.40%)	2.34 (1.15, 4.76)	3.58 (1.48,8.70) **
Never	152(89.9%)	323 (95.6%)	reference	reference
Physical activity				
Sufficient	48 (28.4%)	202 (59.8%)	0.23 (0.15, 0.36)	0.30(0.18,0.50) ***
Insufficient	121(71.6%)	136 (40.2%)	reference	reference
Dietary pattern				
Meat-based	71 (42.0%)	128 (38.0%)	1.44 (0.96–2.17)	1.20 (0.72,2.01)
Processed food based	32 (19.0%)	38 (11.0%)	2.02 (1.20–3.40)	2.11 (1.09,4.08) *
Cereals & Plant based	66 (39.0%)	172 (51.0%)	reference	reference

Tuberculosis				
Yes	16 (9.50%)	11 (3.20%)	2.90(1.35, 6.27)	2.80 (1.10,7.11) *
No	153(90.5%)	327(96.8%)	reference	reference
COPD				
Yes	7 (4.00%)	11 (3.25%)	1.27(0.49, 3.28)	1.52 (0.44,5.27)
No	162(96.0%)	327 (96.5%)	reference	reference
Family history of cancer				
Yes	6 (3.50%)	19 (5.60%)	0.62 (0.24, 1.58)	0.93(0.28, 3.01)
No	163(96.5%)	319 (94.4%)	reference	reference

Note: COPD: chronic obstructive pulmonary diseases, * $p < 0.05$; ** $p < 0.01$; *** $p < 0.001$

Supplementary Table S3. Multivariable conditional logistic regression model for lung cancer risk factors among never-smokers

**Variables**	**Category**	**Cases (%)**	**Controls (%)**	**COR (95% CI)**	**AOR (95% CI)**
Education	Primary	108 (37.11)	256 (38.5)	0.51 (0.35–0.73)	0.59 (0.38–0.91) *
	Secondary & above	52 (17.87)	226 (33.98)	0.24 (0.15–0.38)	0.48 (0.27–0.87) *
	No formal education	131 (45.02)	183 (27.52)	Reference	Reference
Wealth	Low income	155 (53.26)	184 (27.67)	4.60 (3.00–7.04)	3.12 (1.83–5.32) ***
	Medium income	85 (29.21)	244 (36.69)	2.02 (1.28–3.17)	1.59 (0.92–2.74)
	High income	51 (17.53)	237 (35.64)	Reference	Reference
HH solid fuel use [a]	Solid-fuel user	211 (72.51)	329 (49.47)	3.11 (2.23–4.33)	1.93 (1.30–2.86) **
	Nonsolid-fuel user	80 (27.49)	336 (50.53)	Reference	Reference
Occupational ex [b]	Ever	31 (10.65)	50 (7.52)	1.43 (0.83–2.46)	1.37 (0.70–2.69)
	Never	260 (89.35)	615 (92.48)	Reference	Reference
SHS [c]	Ever	27 (9.28)	33 (4.96)	1.95 (1.14–3.36)	3.57 (1.80–7.08) ***
	Never	264 (90.72)	632 (95.04)	Reference	Reference
Physical activity	Sufficient	71 (24.4)	369 (55.49)	0.26 (0.19–0.36)	0.31 (0.21–0.45) ***
	Insufficient	220 (75.6)	296 (44.51)	Reference	Reference
Dietary pattern	Meat-based	125 (42.96)	253 (38.04)	1.34 (0.99–1.82)	1.15 (0.79–1.67)
	Processed based	50 (17.18)	74 (11.13)	1.98 (1.30–3.00)	1.58 (0.95–2.63)
	Cereals & plant based	116 (39.86)	338 (50.83)	Reference	Reference
Tuberculosis	Yes	28 (9.62)	23 (3.46)	2.73 (1.51–4.91)	3.32 (1.62–6.81) **
	No	263 (90.38)	642 (96.54)	Reference	Reference
COPD [d]	Yes	13 (4.47)	27 (4.06)	1.00 (0.51–1.97)	1.95 (0.85–4.47)
	No	278 (95.53)	638 (95.94)	Reference	Reference
Family Ca [e]	Yes	19 (6.53)	27 (4.06)	1.48 (0.79–2.77)	2.11 (0.94–4.71)
	No	272 (93.47)	638 (95.94)	Reference	Reference

Note: [a] household solid fuel use, [b] occupational exposure, [c] secondhand smoke, [d] chronic obstructive pulmonary diseases, [e] family history of cancer, * $p < 0.05$; ** $p < 0.01$; *** $p < 0.001$.

Supplementary Table S4.1: Leave-one-out multivariable conditional logistic regression model for lung cancer risk factors beyond smoking in Ethiopia

		**AOR with 95% CI**			
	**Variable**	**SHS [a]**	**Smoking**	**HH solid fuel use**	**Occupational exposure**
		**Model 1**	**Model 2**	**Model 3**	**Model 4**
Education	Secondary & above	0.59 (0.39– 0.87) **	0.54 (0.37– 0.80) **	0.51 (0.34– 0.76) **	0.54 (0.36– 0.80) **
	Primary (1-8)	0.55 (0.34– 0.90) *	0.45 (0.28– 0.74) **	0.42 (0.26– 0.69) **	0.48 (0.29– 0.79) **
	No formal education	Reference	Reference	Reference	Reference
Wealth	Low	2.26 (1.44– 3.55) ***	2.04 (1.30– 3.18) **	2.46 (1.56– 3.86) ***	2.16 (1.38– 3.40) **
	Medium	1.11 (0.70– 1.75)	1.07 (0.69– 1.68)	1.14 (0.72– 1.79)	1.08 (0.68– 1.70)
	High	Reference	Reference	Reference	Reference
Household solid fuel use	Solid-fuel user	1.74 (1.24– 2.45) **	1.73 (1.23– 2.43) **	—	1.77 (1.25– 2.51) **
	Nonsolid-fuel user	Reference	Reference	Reference	Reference
Occupational exposure [a]	Ever	2.10 (1.18– 3.74) *	1.90 (1.07– 3.36) *	1.82 (1.03– 3.23) *	—
	Never	Reference	Reference	Reference	Reference
Smoking	Ever	4.21 (2.40– 7.36) ***	—	3.81 (2.18– 6.69) ***	3.94 (2.24– 6.93) ***
	Never	Reference	Reference	Reference	Reference
Secondhand smoke	Ever	—	3.12 (1.75– 5.53) ***	2.65 (1.47– 4.76) **	2.88 (1.62– 5.13) ***
	Never	Reference	Reference	Reference	Reference
Physical activity	Sufficient	0.33 (0.23– 0.46) ***	0.29 (0.20– 0.40) ***	0.29 (0.21– 0.41) ***	0.31 (0.22– 0.44) ***
	Insufficient	Reference	Reference	Reference	Reference
Dietary pattern	Meat-based	1.39 (0.99– 1.95)	1.33 (0.95– 1.86)	1.40 (1.00– 1.96) *	1.32 (0.94– 1.86)
	Processed food based	1.66 (1.05– 2.60) *	1.72 (1.11– 2.68) *	1.71 (1.09– 2.68) *	1.67 (1.06 –2.63) *

	Cereals & Plant based	Reference	Reference	Reference	Reference
Tuberculosis	Yes	3.12 (1.69– 5.78) ***	3.52 (1.92– 6.45) ***	3.33 (1.78– 6.22) ***	3.20 (1.73– 5.92) ***
	No	Reference	Reference	Reference	Reference
COPD [c]	Yes	1.99 (0.90– 4.44)	2.02 (0.95– 4.29)	1.96 (0.90– 4.25)	1.95 (0.88– 4.32)
	No	Reference	Reference	Reference	Reference
Family History of cancer	Yes	2.36 (1.16– 4.79) *	2.10 (1.03– 4.30) *	1.97 (0.96– 4.07)	2.27 (1.09– 4.73) *
	No	Reference	Reference	Reference	Reference

Note: [a] secondhand smoke, [b] household solid fuel use, [c] COPD: chronic obstructive pulmonary diseases, * $p < 0.05$; ** $p < 0.01$; *** $p < 0.001$

Supplementary Table S4.2: Leave-one-out multivariable conditional logistic regression model for lung cancer risk factors beyond smoking in Ethiopia

		AOR 95% CI			
	**Variable**	**COPD [a]**	**Tuberculosis**	**Dietary pattern**	**Physical activity**
		**Model 5**	**Model 6**	**Model 7**	**Model 8**
Education	Secondary & above	0.52 (0.35– 0.78) **	0.50 (0.34– 0.75) **	0.53 (0.35– 0.79) **	0.58 (0.40– 0.85) **
	Primary (1-8)	0.50 (0.30– 0.83) **	0.52 (0.32– 0.85) **	0.49 (0.30– 0.81) **	0.54 (0.33– 0.88) *
	No formal education	Reference	Reference	Reference	Reference
Wealth	Low	2.27 (1.44– 3.58) ***	2.33 (1.48– 3.67) ***	2.24 (1.43– 3.53) ***	2.70 (1.74– 4.19) ***
	Medium	1.11 (0.70– 1.76)	1.09 (0.69– 1.73)	1.13 (0.72– 1.79)	1.33 (0.85– 2.06)
	High	Reference	Reference	Reference	Reference
Household solid fuel use	Solid-fuel user	1.78 (1.26– 2.52) **	1.81 (1.29– 2.55) **	1.81 (1.28– 2.56) **	2.17 (1.55– 3.04) ***
	Nonsolid-fuel user	Reference	Reference	Reference	Reference
Occupational exposure [a]	Ever	1.88 (1.05– 3.38) *	1.91 (1.07– 3.41) *	1.83 (1.02– 3.28) *	2.07 (1.18– 3.63) *
	Never	Reference	Reference	Reference	Reference
Smoking	Ever	3.93 (2.23– 6.91) ***	4.16 (2.38– 7.26) ***	4.00 (2.26– 7.09) ***	4.81 (2.79– 8.30) ***
	Never	Reference	Reference	Reference	Reference
Secondhand smoke	Ever	2.71 (1.51– 4.85) **	2.61 (1.47– 4.64) **	2.68 (1.51– 4.77) **	2.33 (1.34– 4.03) **
	Never	Reference	Reference	Reference	Reference
Physical activity	Sufficient	0.32 (0.23– 0.46) ***	0.32 (0.23– 0.46) ***	0.30 (0.22– 0.43) ***	—
	Insufficient	Reference	Reference	Reference	Reference
Dietary pattern	Meat-based	1.34 (0.95– 1.88)	1.27 (0.91– 1.78)	—	1.42 (1.03– 1.95) *
	Processed food based	1.68 (1.07– 2.65) *	1.62 (1.03– 2.56) *	—	1.98 (1.29– 3.04) **

	Cereals & Plant based	Reference	Reference	Reference	Reference
Tuberculosis	Yes	3.17 (1.70– 5.91) ***	—	3.03 (1.63– 5.67) ***	3.13 (1.73– 5.69) ***
	No	Reference	Reference	Reference	Reference
COPD	Yes	—	1.91 (0.86– 4.22)	1.91 (0.87– 4.19)	1.59 (0.76– 3.32)
	No	Reference	Reference	Reference	Reference
Family History of cancer	Yes	2.13 (1.03– 4.42) *	2.29 (1.12– 4.69) *	1.97 (0.96– 4.06)	2.01 (0.99– 4.10)
	No	Reference	Reference	Reference	Reference

Note: [a] COPD: chronic obstructive pulmonary diseases, * $p < 0.05$; ** $p < 0.01$; *** $p < 0.001$

Supplementary Table S5: Multivariable logistic regression model for lung cancer risk factors beyond smoking in Ethiopia

	AOR 95% CI [a]		
**Variables**	**Both sexes**	**Male**	**Female**
Education			
Primary	0.70 (0.50–0.97)	0.61 (0.37–1.00)	0.81 (0.49–1.32)
Secondary & above	0.60 (0.38–0.92)	0.48 (0.27–0.88)	0.85 (0.42–1.72)
No formal education	Reference	Reference	Reference
Wealth			
Low	2.35 (1.54–3.58)	2.11 (1.15–3.86)	2.72 (1.43–5.16)
Medium	1.16 (0.78–1.75)	1.29 (0.75–2.23)	1.05 (0.55–2.04)
High	Reference	Reference	Reference
Household solid fuel use			
Solid-fuel user	1.77 (1.28–2.43)	1.45 (0.94–2.25)	2.39 (1.45–3.95)
Nonsolid-fuel user	Reference	Reference	Reference
Occupational Exposure			
Ever	1.36 (0.83–2.24)	1.84 (0.95–3.54)	0.77 (0.33–1.78)
Never	Reference	Reference	Reference
Smoking			
Ever	3.70 (2.29–5.99)	4.98 (2.80–8.86)	1.94 (0.74–5.11)
Never	Reference	Reference	Reference
Secondhand smoke			
Ever	2.20 (1.28–3.80)	1.55 (0.71–3.36)	2.88 (1.28–6.49)
Never	Reference	Reference	Reference
Physical activity			
Sufficient	0.32 (0.24–0.44)	0.33 (0.21–0.52)	0.31 (0.20–0.48)
Insufficient	Reference	Reference	Reference
Dietary pattern			
Meat based	1.35 (0.99–1.85)	1.60 (1.02–2.50)	1.18 (0.74–1.87)
Processed food-based	1.91 (1.25–2.93)	1.76 (0.95–3.28)	2.18 (1.17–4.06)
Cereals & plant-based	Reference	Reference	Reference

Tuberculosis			
Yes	3.91 (2.18–7.03)	3.69 (1.63–8.37)	4.24 (1.75–10.26)
No	Reference	Reference	Reference
COPD [b]			
Yes	1.78 (0.86–3.66)	2.26 (0.85–6.02)	1.31 (0.42–4.09)
No	Reference	Reference	Reference
Family History of Cancer			
Yes	1.55 (0.80–3.00)	3.21 (1.27–8.06)	0.61 (0.20–1.88)
No	Reference	Reference	Reference

Note: [a] adjusted odds ratio with 95% confidence interval, **[b]** chronic obstructive pulmonary diseases

Supplementary Table S6. Multivariable firth logistic regression model for lung cancer risk factors beyond smoking in Ethiopia

	AOR (95% CI)		
**Variable**	**Both Sexes**	**Male**	**Female**
Education			
Secondary & above	0.70(0.50– 0.98)	0.61(0.37– 1.00)	0.81(0.49– 1.32)
Primary (1-8)	0.60(0.39– 0.93)	0.48(0.27– 0.88)	0.85(0.42– 1.72)
No formal education	Reference	Reference	Reference
Wealth			
Low	2.31(1.52– 3.51)	2.11(1.15– 3.86)	2.72(1.43– 5.16)
Medium	1.16(0.78– 1.73)	1.29(0.75– 2.23)	1.05(0.55– 2.04)
High	Reference	Reference	Reference
Household solid fuel use			
Solid-fuel user	1.75(1.27– 2.40)	1.45(0.94– 2.25)	2.39(1.45– 3.95)
Nonsolid-fuel user	Reference	Reference	Reference
Occupational exposure			
Ever	1.36(0.83– 2.22)	1.84(0.95– 3.54)	0.77(0.33– 1.78)
Never	Reference	Reference	Reference
Smoking			
Ever	3.60(2.24– 5.79)	4.98(2.80– 8.86)	1.94(0.74– 5.11)
Never	Reference	Reference	Reference
Secondhand smoke			
Ever	2.18(1.27– 3.73)	1.55(0.71– 3.36)	2.88(1.28– 6.49)
Never	Reference	Reference	Reference
Physical activity			
Sufficient	0.33(0.24– 0.45)	0.33(0.21– 0.52)	0.31(0.20– 0.48)
Insufficient	Reference	Reference	Reference
Dietary pattern			
Meat-based	1.34(0.98– 1.83)	1.60(1.02– 2.50)	1.18(0.74– 1.87)
Processed food based	1.90(1.24– 2.89)	1.76(0.95– 3.28)	2.18(1.17– 4.06)
Cereals & Plant based	Reference	Reference	Reference

Tuberculosis			
Yes	3.80(2.13– 6.78)	3.69(1.63– 8.37)	4.24(1.75– 10.26)
No	Reference	Reference	Reference
COPD [a]			
Yes	1.78(0.87– 3.63)	2.26(0.85– 6.02)	1.31(0.42– 4.09)
No	Reference	Reference	Reference
Family history of cancer			
Yes	1.53(0.80– 2.95)	3.21(1.27– 8.06)	0.61(0.20– 1.88)
No	Reference	Reference	Reference

Note: [a] COPD: chronic obstructive pulmonary diseases
